# Exploring whether relationship mobility and goal congruence in travel groups enhance tourists’ pro-environmental behaviors: new insights from social cognitive theory

**DOI:** 10.3389/fpsyg.2025.1681160

**Published:** 2025-11-03

**Authors:** Junxi Gao, Weiwei Deng

**Affiliations:** School of Tourism and Culture Industry, Sichuan Tourism University, Chengdu, China

**Keywords:** social cognitive theory, goal congruence, relationship mobility, low-cost pro-environmental behaviors, high-cost pro-environmental behaviors

## Abstract

This study addresses the research gap in group-level pro-environmental behaviors by establishing a social cognitive theoretical model within travel groups. Employing a quantitative design and structural equation modeling, it examines key antecedents, the mediating role of relationship mobility, and the moderating effect of Goal Congruence. Findings reveal that environmental emotion/values, impression management, and relationship mobility significantly promote low-cost pro-environmental behaviors, while only environmental emotion/values positively affects high-cost behaviors. Relationship mobility mediates the effects of environmental emotion/values and impression management on low-cost behaviors, and the effect of impression management on high-cost behaviors. Goal Congruence moderates the effects of environmental emotion/values and impression management on both low-cost and high-cost behaviors (except environmental emotion/values’ direct effect on high-cost). While limited by its focus on travel groups and self-reported data, the research significantly advances understanding by shifting to the group level in tourism, empirically validating distinct pathways for different behavior costs, and highlighting relationship mobility and Goal Congruence’s critical roles. It offers practical strategies for leveraging group dynamics.

## 1 Introduction

In the era of globalization, pro-environmental behavior (PEB) serves as a cornerstone for organizational sustainability initiatives and environmental protection (Biswas et al., 2022). With the recovery and development of the tourism industry, group travel has re-emerged as a mainstream choice due to its convenience and efficiency. However, its scale effect also exacerbates environmental pressures on tourist destinations (Font et al., 2021). Within this context, understanding and promoting tourists’ PEB is widely recognized as pivotal to achieving sustainable tourism (Zhang et al., 2023; Han et al., 2023). Recent research in tourism has increasingly focused on tourists’ pro-environmental behavior (PEB). Studies have primarily examined the influence of internal psychological factors, such as awe (Wang et al., 2025), eco-literacy and net-zero commitment (Salem et al., 2025), environmental values, and environmental sensitivity (Elsamen et al., 2025) on PEB. External contextual factors have also been explored, including relaxing tourism activities (Su et al., 2025) and environmental (social) cues (Cheng et al., 2024) in eliciting PEB. Furthermore, some scholars have developed comprehensive models integrating both internal and external factors to investigate tourists’ intentions toward pro-environmental behavior (Zhang et al., 2025). Yet, it has largely overlooked the potential impact of the interaction between tourists’ “self” and their immediate situational context on PEB. The interrelationships between tourists’ internal states and external environments remain underexplored. Furthermore, a critical research perspective—the micro-level social dynamics within temporary tourism groups—has been largely neglected (Font et al., 2021). This oversight constrains a comprehensive understanding of the formation mechanisms of PEB in group settings.

Social cognitive theory provides a robust theoretical framework for integrating individual behavior with environmental factors (Bandura, 2001, 2012). Unfortunately, tourism research from this perspective remains scarce and has often simplistically equated “environmental” factors with static physical or socio-cultural contexts, thereby failing to capture the emergent and dynamic group interaction processes within temporary collectives. In essence, the group tourism experience is inherently social (Yuki and Schug, 2020). Within such transient systems, the relational adjustments members make to maintain harmony (relational mobility) and the consensus formed to achieve shared experiential goals (goal congruence) constitute the immediate and core social environment that shapes individual behavior (Lundin, 2007; Schaffer, 2007). Consequently, the direct applicability of findings derived from studies focused on individuals or stable social relationships to this dynamic context remains questionable.

Although recent research has begun to focus on social factors. For instance, relational mobility has been shown to promote pro-environmental behavior through impression management (Dong et al., 2023a; Dong et al., 2023b) these studies often presuppose a relatively stable social context. They have yet to adequately explore its unique operational mechanisms within the transiently formed social structures of tourist groups. Similarly, while the significance of goal congruence in long-term organizations has been well established (Schaffer, 2007), whether and how it moderates individual behavior within short-term, leisure-oriented tourist groups remains largely unexplored. More critically, seminal research on behavioral cost posits that the underlying drivers of high-cost and low-cost pro-environmental behaviors may be fundamentally distinct (Abrahamse and Steg, 2011). However, existing studies exploring group dynamics have predominantly overlooked this crucial boundary condition, resulting in a potentially oversimplified understanding of behavioral motivations.

To address these research gaps, this study employs temporary tourist groups as its empirical context and aims to achieve three core objectives. First, it seeks to develop an integrated model grounded in social cognitive theory, incorporating personal factors (environmental emotion, environmental values, impression management), group-level social environmental factors (relational mobility), and behavioral outcomes (high-cost and low-cost pro-environmental behaviors) into a unified framework. Second, it aims to empirically test the moderating role of goal congruence within this model, elucidating when and how shared group goals amplify or attenuate the influence of personal factors on behavior. Third, the study delves into the mediating mechanism of relational mobility between personal factors and pro-environmental behaviors, with a specific focus on examining its differential functioning across high-cost and low-cost behavioral contexts. By doing so, this research not only seeks to extend the application boundaries of social cognitive theory within tourism research but also aims to offer profound theoretical insights for tourism managers seeking to precisely foster different tiers of environmental behaviors by optimizing group dynamics.

## 2 Theoretical background

### 2.1 Pro-environmental behavior

Based on the literature, the definition of PEB vary significantly (Lange, 2024; Gatersleben, 2023; Lange and Dewitte, 2019; Stern, 2000). However, PEB is generally referred to as “environmentally friendly behavior,” “environmentally responsible behavior,” or “green behavior,” and is characterized by actions where individuals consciously seek to minimize the negative impacts of their behavior on the natural and built environment (Kollmuss and Agyeman, 2002). It includes actions that benefit the environment or minimize harm to it (Stern, 2000; Han et al., 2009; Halpenny, 2010). From an impact-oriented perspective, PEB is defined as behavior that provides relatively favorable outcomes for the natural environment. From an intent-based perspective, PEB refers to actions performed based on presumed environmental benefits, that is, behaviors driven by the goal or prospect of protecting the environment (Lange, 2024).

PEB is the result of various antecedents and factors that stimulate such behaviors among individuals (Adam et al., 2021; Fauzi et al., 2023; Han, 2015). Extensive academic research has examined the antecedents of PEB, identifying cultural influences, values, and the costs and convenience of PEBs as significant determinants of their adoption (Zhang et al., 2016; Verplanken and Orbell, 2022; Shang et al., 2023). Progressing research on PEB has expanded to include various aspects such as individual psychological motivations (Davari et al., 2024; Kronrod et al., 2023; Ribeiro et al., 2023), social norms (Helferich et al., 2023; D’Arco et al., 2023), and policy interventions (Alt et al., 2024). Current studies on PEB often focus on specific behaviors, such as farmers adopting eco-friendly agricultural practices (Xie and Huang, 2021), employees’ green workplace behaviors (Zacher et al., 2023), and Generation Z’s pro-environmental travel behaviors (Ribeiro et al., 2023).

Behavioral cost serves as a pivotal variable in explaining the attitude-behavior consistency gap. According to the “low-cost hypothesis,” the cost of alternative actions not only directly shapes behavior—as posited by traditional rational choice theory—but also moderates the effect of attitudes on the corresponding behavior. In a broad sense, every behavior is associated with a “cost,” which individuals may perceive as high or low (Diekmann and Preisendörfer, 2003). A behavior is perceived as high-cost if it entails significant perceived sacrifices, such as intolerably prolonged travel time or reduced comfort. In contrast, low-cost behaviors—including one-time investments (e.g., resetting the default settings of a thermostat) and simple daily tasks like turning off lights when leaving a room—are easier to implement than their high-cost counterparts. Grounded in this action-cost perspective, the present study categorizes pro-environmental behaviors into high-cost and low-cost types. High-cost pro-environmental behaviors refer to those requiring considerable effort, time, or financial resources, whereas low-cost behaviors involve minimal sacrifices or expenses (Abrahamse and Steg, 2011).

### 2.2 Social cognitive theory

Proposed by the American psychologist Bandura in the 1970s, SCT posits that individual actions result from the interaction of three factors: environment, individual cognition, and behavior. This theory provides a “cognition-behavior-environment” framework for understanding and predicting human activities (Cai and Shi, 2020). The environment encompasses external factors beyond individual attributes that influence behavior. These include physical (e.g., work equipment and availability of necessary resources) and social environments (e.g., family, friends, colleagues, and relational atmosphere) (Bandura, 1979). Some studies have suggested that the environmental factors within SCT include external models, guidance, feedback, rewards, and opportunities for self-assessment. By contrast, personal factors are internal and include cognition, goals, self-efficacy, social comparisons, outcome expectations, and attributions (Schunk and DiBenedetto, 2020). This theory is renowned for its research on observational learning, self-efficacy, and reciprocal determinism, with experiments demonstrating that individuals can learn new behaviors by observing others and the consequences of their actions. If a behavior is rewarded (either positively or negatively), it is more likely to be imitated; conversely, if it is punished, the likelihood of imitation decreases (Nickerson, 2024).

Historically, social cognitive theory has been frequently employed to predict offline consumer behaviors, such as repurchase behavior and sustainable consumption (Phipps et al., 2013; Kursan Milaković, 2021). Subsequently, its application has progressively expanded into the tourism and hospitality sectors (Wang et al., 2019). Within the field of tourism research, the theory has been utilized to explain consumer behaviors and responses across diverse contexts. For instance, in rural tourism settings, outcome expectations have been found to motivate tourists’ intention to use travel applications (Lu et al., 2015); in sustainability initiatives, it has been applied to examine the motivations underlying sustainable tourism (Font et al., 2016); in digital tourism contexts, technological service innovation has been shown to influence tourists’ revisit behavior (Preko et al., 2023); and in hotel accommodation scenarios, technostress has been demonstrated to affect visitor satisfaction and pleasure (Lee et al., 2023). More recent scholarly focus has extended to areas such as children’s learning processes within the context of family travel (Li et al., 2024) and understanding how eco-friendly hotels influence customers’ green patronage intention and green word-of-mouth (Nosrati et al., 2025). Collectively, these studies underscore the applicability of social cognitive theory in predicting consumer decision-making. Nevertheless, its application in research on pro-environmental behaviors within the tourism domain remains relatively underutilized.

According to Social Cognitive Theory (SCT), individuals are not merely passive recipients of external influences but rather active agents who selectively choose and modify their surroundings to facilitate learning. Therefore, compared to other theories widely used in tourism research, SCT provides a more comprehensive framework for understanding tourist behaviors, including pro-environmental actions. The concept of triadic reciprocal determinism among environmental, behavioral, and personal factors forms the theoretical foundation of this study, offering a structured approach for data analysis and result interpretation.

Grounded in this perspective, the present study adopts an SCT lens to investigate the formation mechanisms of pro-environmental behaviors among tourists within temporarily formed travel groups, thereby extending the theory to a novel empirical context. Specifically, environmental factors are manifested as relational mobility within the social environment, while individual factors correspond to tourists’ environmental emotions, environmental values, and personal impression management. These elements collectively shape tourists’ engagement in pro-environmental behaviors.

### 2.3 Environmental emotions

Emotions are considered to be one of the factors that influence behavior. Anticipated positive and negative emotions can act as determinants of behavioral intentions (Perugini and Bagozzi, 2001). Environment-related behaviors encompass pro-environmental actions such as recycling, energy conservation, and participation in environmental protests, and environmentally harmful actions such as driving cars, flying, overconsumption, and engaging in leisure activities that are detrimental to the environment. Emotions triggered by these behaviors, objects, individuals related to them, or the natural environment itself can be viewed as EE (Kals and Müller, 2012). Environmental conservation involves multiple aspects including morality, responsibility, environmental risks, and natural sentiments. Thus, the emotions related to environmental protection can be classified into three categories: 1. Moral Emotions: These are emotional responses related to an individual’s conscience and grounded in moral principles. 2. Social Emotions: These emotions arise from individuals’ perceptions of social actions such as commitment to pollution control or environmental conservation. 3. Ecological Emotions: These include concerns about climate change and feelings about ecological care (Kao and Du, 2020). Individuals’ concerns about the environment influence their emotional responses, which in turn predict their behavior. Based on these insights, this study proposes the following hypothesis:

*H1a:* EE have a positive effect on relational mobility (RM).

*H1b:* EE positively impact low-cost PEBs.

*H1c:* EE positively impact high-cost PEBs.

### 2.4 Environmental values

Value theory provides guidance for individuals’ social lives by shaping their beliefs, attitudes, and behaviors (Schwartz, 1994). For individuals, EV direct their choices toward green living (Guagnano et al., 1995). Stern (1992) Value-Belief-Norm theory integrates value theory with norm activation theory, positing that PEB is driven by personal norms, which, in turn, are influenced by EV (Lim, 2024). Stern (2000) categorized EV into three dimensions: egoistic, altruistic, and biospheric (Schwartz, 1994).

Specifically, egoistic, altruistic, and biospheric values influence trust, interaction frequency, and relational closeness among individuals, thereby enhancing RM. EV help individuals to identify like-minded partners in dynamic social environments, allowing them to flexibly adjust and select relationships, thereby improving the stability and sustainability of social networks (Stern, 2000; Guagnano et al., 1995). Based on this analysis, we propose the following hypotheses:

*H2a:* EV positively impact RM.

Values act as drivers of attitudes and behaviors. Egoistic, altruistic, and biospheric values directly influence low-cost PEBs in daily life, such as reducing water usage and sorting waste. These behaviors involve minimal costs and are easy to integrate into daily routines; thus, they remain consistently guided by values (Fulton et al., 1996). Based on this analysis, we propose the following hypothesis:

*H2b:* EV positively impact low-cost PEBs.

Guided by values, egoistic, altruistic, and biospheric values encourage individuals to invest resources in high-cost PEBs such as purchasing eco-friendly products or participating in long-term environmental projects (Rokeach, 1973; Kaltenborn et al., 2002). Individuals with strong EV are more willing to bear higher behavioral costs to reduce their environmental impact. This investment demonstrates their commitment to the environment and enhances their social approval. Based on this analysis, we propose the following hypothesis:

*H2c:* EV positively impact high-cost PEBs.

### 2.5 Impression management

IM refers to the strategies individuals use to shape how others perceive them in a desired manner (Goffman, 1959). People tend to employ various tactics to attract and maintain valuable interpersonal relationships, thereby distinguishing themselves from their competitors. Individuals can enhance their attractiveness by highlighting desirable traits such as strength, intelligence, and generosity (Barclay, 2016). Moreover, as their sense of belonging increases, their supportive and helpful behaviors toward partners also increase (Yamada et al., 2015). Accordingly, we propose the following hypothesis:

*H3a*: IM positively influences RM.

As a self-regulatory behavior management strategy, IM is effective in high-investment contexts and in low-cost situations (Wooten and Reed, 2000; Leary and Kowalski, 1990). When individuals aim to exhibit behaviors that align with social norms, they actively engage in low-cost PEBs (e.g., reducing plastic use and sorting waste) to demonstrate their environmental concerns and maintain a positive social image (Amaral et al., 2019; Schlenker, 1980). Accordingly, we propose the following hypothesis:

*H3b:* IM positively influences low-cost PEBs.

According to Goffman’s dramaturgical theory, individuals adopt IM strategies in specific contexts to shape how others perceive them (Goffman, 1959). In unfamiliar settings such as temporarily formed travel groups, people often wish to project a positive image aligned with social norms to enhance others’ perceptions. For high-cost PEBs (e.g., participating in environmental volunteer activities or committing to long-term environmental projects), the motivation for IM encourages individuals to choose these behaviors, even when they involve resource or cost expenditures, to gain social approval and enhance their self-image (Hartwell et al., 2019; Suen and Hung, 2024). Accordingly, we propose the following hypothesis:

*H3c:* IM positively influences high-cost PEBs.

### 2.6 Relational mobility

Within social structures, some individuals have abundant opportunities to choose new partners, allowing them to freely establish and modify relationships and embed themselves firmly within their social networks. Conversely, other individuals have fewer opportunities or lack the necessity to take risks when breaking away from existing relationships to seek new social connections (Yuki and Schug, 2020). With the globalization of human societies, RM has become an increasingly significant concept for understanding how individuals are embedded in social systems (Dong et al., 2023a; Dong et al., 2023b; Thomson et al., 2018; Yuki et al., 2007).

Yuki and Schug (2020) proposed that the level of RM in society profoundly influences individuals’ thought patterns and behaviors. This impact is not confined to the establishment and maintenance of interpersonal relationships but extends deeply into cognitive processes. Moreover, Dong et al. (2023a) and Dong et al. (2023b) revealed that RM, as an external environmental factor, can significantly affect individuals’ PEBs through subtle psychological mechanisms. Based on these insights, we propose the following hypotheses:

*H4a:* RM positively influences low-cost PEBs.

*H4b:* RM positively influences high-cost PEBs.

Schug et al. (2010) highlight that high RM encourages individuals to strengthen and cultivate ideal interpersonal relationships, thereby promoting opportunities for growth and self-expansion. IM, as a key measure of self-serving motivation (Liao et al., 2022), enhances individuals’ PEBs (Zhang et al., 2019). According to the IM theory, this process involves self-regulation, wherein individuals actively adjust their behavior to project a positive image onto others (Wooten and Reed, 2000). Based on this, we propose the following hypotheses:

*H5a:* RM mediates the relationship between IM and high-cost PEBs.

*H5b:* RM mediates the relationship between EV and high-cost PEBs.

*H5c:* RM mediates the relationship between EE and high-cost PEBs.

*H5d:* RM mediates the relationship between IM and low-cost PEBs.

*H5f:* RM mediates the relationship between EV and low-cost PEBs.

*H5e:* RM mediates the relationship between EE and low-cost PEBs.

### 2.7 Goal congruence

Individuals regulate their behavior based on their set goals, and the dispersion of goal perceptions among team members can reduce interdependence within the team, thereby hindering collaboration (Lundin, 2007). Therefore, achieving GC is crucial. From the perspective of organizational goal management, one critical factor in ensuring the performance of organizations, teams, and individuals is the formulation and implementation of clear goals (Mento et al., 1987). For teams to operate efficiently and positively, a well-designed goal must earn the recognition and commitment of team members and stimulate their motivation and enthusiasm. Such goals promote alignment between team members and their leaders and among team members (Schaffer, 2007).

EE represent individuals’ affective attitudes toward the environment, whereas low-cost and high-cost PEBs involve varying levels of resource commitment. When individuals experience environmental protection-related emotions and their personal goals align with these behaviors, GC amplifies the influence of emotions on pro-environmental actions. Within temporary travel groups, the alignment of goals among team members may encourage such behaviors. Thus, individuals are more likely to engage in pro-environmental actions, regardless of whether these actions are of low or high cost. Based on these analyses, we propose the following hypotheses:

*H6a:* GC strengthens the influence of EE on low-cost PEBs.

*H6b:* GC strengthens the influence of EE on high-cost PEBs.

When personal values align with environmental protection goals, this congruence increases the likelihood of individuals exhibiting behaviors consistent with their EV. High-value congruence enhances the propensity for PEBs regardless of whether they involve minimal resources (low cost) or significant investments (high cost). Based on these insights, we propose the following hypotheses:

*H6c*: GC strengthens the influence of EV on low-cost PEBs.

*H6d:* GC strengthens the influence of EV on high-cost PEBs.

RM refers to an individual’s ability to freely form relationships within social networks. Within groups, GC fosters a shared belief in environmental protection among members, motivating them to exhibit PEBs under group pressure or through conformity. Thus, GC enhances the impact of RM on PEBs, whether at a low or high cost.

*H6e:* GC strengthens the influence of RM on low-cost PEBs.

*H6f:* GC strengthens the influence of RM on high-cost PEBs.

Current research has tended to evaluate cognitive and emotional factors separately (Tian and Liu, 2022). For example, the Theory of Planned Behavior primarily focuses on cognitive aspects, such as attitudes, subjective norms, and perceived behavioral control, without adequately considering the influence of emotions (Conner, 2020). This separation renders the theoretical explanations of the actual behavior incomplete and insufficiently systematic. To address this gap, this study employed SCT to integrate cognitive and emotional factors to construct a comprehensive theoretical framework for PEBs encompassing rational and emotional dimensions.

**Figure 1:**
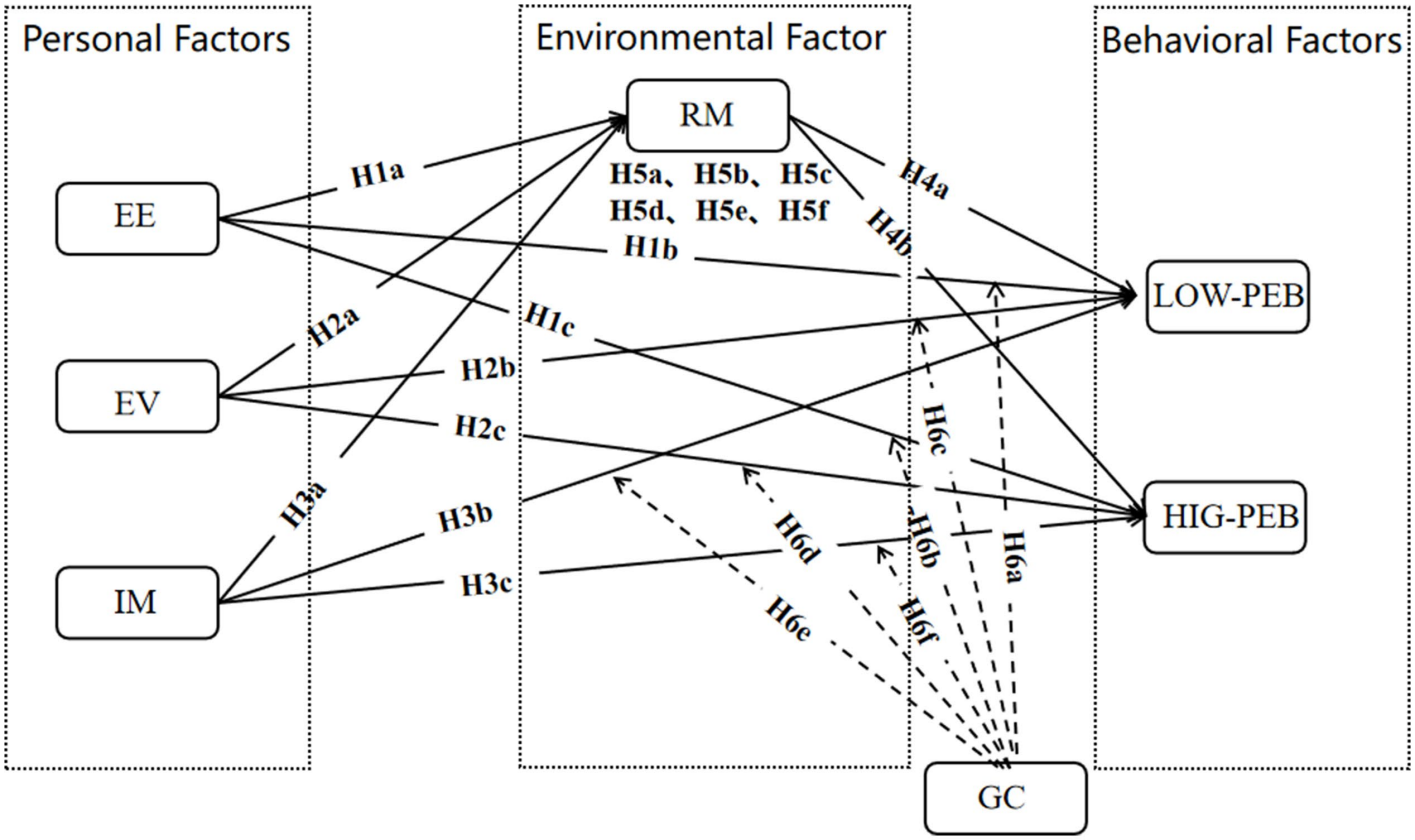
Conceptual model based on social cognitive theory.

By leveraging SCT, this study incorporated internal and external factors to build a holistic framework for understanding PEBs (Figure 1). This integrated approach provides a more robust explanation of such actions.

## 3 Sample and data collection

### 3.1 Measurement

The scales used in this study were rigorously validated before being modified and applied to assess the constructs. The questionnaire was divided into two parts: basic demographic information and the measurement scales. The basic information section included gender, age, education level, occupation type, and monthly income. In the measurement section, pro-environmental behaviors were categorized into high-cost and low-cost types. Tourists engaging in high-cost pro-environmental behaviors perceive greater sacrifices of personal resources and demonstrate stronger self-regulation in the absence of external monitoring compared to those performing low-cost behaviors. To measure the distinctions and impacts between these behavioral types, a five-point Likert scale comprising 41 items across six conceptual domains was developed. Specifically, the pro-environmental behavior scale was adapted from Abrahamse and Steg (2011), consisting of two latent variables: high-cost pro-environmental behavior (e.g., donating to conservation initiatives, intervening against environmentally damaging behaviors, persuading companions to protect natural habitats) and low-cost pro-environmental behavior (e.g., practicing low-impact tourism, proper waste disposal, prioritizing eco-friendly products). These were measured through 10 total items. The environmental emotion scale, adapted from Kao and Du (2020) and Kals and Müller (2012), comprised six items (e.g., “I am amazed by the beauty of nature around me,” “I feel awe toward nature almost every day”). The environmental value scale, derived from Stern (2000), comprised five items (e.g., “The natural environment has value equal to that of humans,” “To protect natural resources and the environment, humans should not develop more resources and land”). The IM scale, adapted from the work of Ingold et al. (2016) and Goffman (1959), comprised four items (e.g., “I care about how others perceive me,” “I want to appear better in the eyes of others”). The RM Scale, based on Yuki et al. (2007) RM Scale, included 12 items (e.g., “I have many opportunities to meet new people,” “It is common to talk to strangers”). Finally, to measure GC within the group, four items were adapted from De Clercq et al. (2011) (e.g., “I share similar views with those around me on how PEBs should be carried out,” “I align with others around me on issues related to environmental protection”).

### 3.2 Data collection and sample

In February 2024, the research team conducted a preliminary investigation in the Jiuzhaigou National Nature Reserve, located in the Aba Tibetan and Qiang Autonomous Prefectures of Sichuan Province. With the assistance of local tourism companies, the team surveyed *ad hoc* travel groups by distributing 200 questionnaires and collecting 181 valid responses. Following exploratory data analysis and reliability and validity tests on the preliminary data, items with factor loadings below the standard threshold of 0.6 were removed, ensuring the scientific rigor of the formal survey. From March to April 2024, the research team conducted a formal survey in the same location, distributing 680 questionnaires. Of these, 612 were returned, with 4 deemed invalid, resulting in 608 valid responses. The respondents were predominantly from various Chinese cities. Among them, 46.8% were men (284 participants) and 53.2% were women (324 participants). The overall response rate was 89.4%. All participants gave their informed consent for inclusion before they participated in the study.

### 3.3 Empirical study

Demographic analysis of the sample, as shown in Table 1.

**Table 1:** Demographic profile of respondents.

Items	Type	Number	Percentage
Gender	Male	284	46.8%
	Female	324	53.2%
Age	0–24	218	35.8%
	25–35	200	32.9%
	35–45	135	22.2%
	45+	55	9.1%
Education	Less than high school	12	1.9%
	Junior college graduate	264	43.42%
	College graduate	288	47.37%
	Above college graduate	44	7.2%

## 4 Measurement scales

### 4.1 Evaluation of the measurement model

This study employed the Partial Least Squares (PLS) method for data analysis using the Smart PLS software (version 4.0). As shown in Table 2, the study analyzed the reliability and validity of each variable, and the external factor loadings. Additionally, demographic variables such as gender, age, education, occupation, and frequency of use were considered as potential influences on the experimental results. Therefore, these variables were included as control variables in the analysis.

**Table 2:** Results of the measurement model.

Latent variables	Items	Loadings	Cronbach’s *α*	CR	AVE
EE	EE1	0.764	0.827	0.886	0.660
	EE2	0.783			
	EE3	0.866			
	EE4	0.833			
EV	EV1	0.812	0.862	0.901	0.648
	EV2	0.792			
	EV3	0.845			
	EV4	0.804			
	EV5	0.859			
GC	GC1	0.879	0.884	0.928	0.811
	GC2	0.893			
	GC3	0.93			
IM	IM1	0.787	0.875	0.914	0.728
	IM2	0.847			
	IM3	0.887			
	IM4	0.888			
RM	RM1	0.887	0.808	0.886	0.722
	RM2	0.823			
	RM3	0.848			
LOW-PEB	LOW-PEB1	0.748	0.799	0.86	0.552
	LOW-PEB2	0.763			
	LOW-PEB3	0.723			
	LOW-PEB4	0.719			
	LOW-PEB5	0.762			
HIG-PEB	HIG-PEB1	0.757	0.795	0.865	0.615
	HIG-PEB2	0.748			
	HIG-PEB3	0.844			
	HIG-PEB4	0.785			

The reliability and validity of the 608 collected questionnaires were examined and the results are presented in Tables 2–4. In this study, reliability was primarily measured using Cronbach’s *α* coefficient and composite reliability (CR). The results indicate that the Cronbach’s α coefficients for the seven latent variables (environmental emotion, EV, IM, RM, GC, low-cost PEB, and high-cost PEB) all exceeded the commonly accepted threshold of 0.7. Similarly, the CR values for all these variables were >0.7. These findings demonstrate that the scales designed for this study exhibit good internal consistency and that the data are highly reliable.

The validity tests conducted in this study encompassed convergent and discriminant validity. The average variance extracted values for EE, EV, IM, RM, GC, low-cost PEB, and high-cost PEB exceeded 0.5. This indicates that the scales and data exhibited good convergent validity (Hair et al., 2014). To comprehensively assess the discriminant validity of the measurement model, this study employed both the Fornell-Larcker criterion (Fornell and Larcker, 1981) and the Heterotrait-Monotrait Ratio (HTMT) of correlations (Henseler et al., 2015). As shown in Table 3, the square roots of the Average Variance Extracted (AVE) for each construct (values on the diagonal) are greater than the correlations with other constructs (values below the diagonal), indicating adequate discriminant validity. Furthermore, as presented in Table 4, all HTMT values between constructs are below the conservative threshold of 0.85.

**Table 3:** Discriminant validity of the measurement model.

	LOW-PEB	IM	EV	EE	RM	HIG-PEB
LOW-PEB	**0.743**					
IM	0.435	**0.853**				
EV	0.537	0.572	**0.805**			
EE	0.556	0.442	0.604	**0.812**		
RM	0.072	0.155	0.122	0.082	**0.941**	
HIG-PEB	0.424	0.402	0.588	0.77	0.121	**0.784**

Note: The diagonals represent the average variance extracted and the lower cells represent the squared correlations among the constructs.

**Table 4:** Discriminant validity assessment (Heterotrait-Monotrait ratio, HTMT).

	LOW-PEB	IM	EV	EE	RM	HIG-PEB
LOW-PEB						
IM	0.518					
EV	0.603	0.633				
EE	0.704	0.516	0.702			
RM	0.383	0.309	0.312	0.326		
HIG-PEB	0.524	0.463	0.709	0.725	0.251	

### 4.2 Evaluation of the structural model

Using SmartPLS 4.0 software, we conducted structural model testing via the Bootstrap method (*N* = 5,000 samples) (Chin, 2010). The evaluation of the structural model involved several metrics, including the significance of path coefficients, f^2^, R^2^, Q^2^, and goodness of fit (GoF) (Hair et al., 2014).

**Figure 2:**
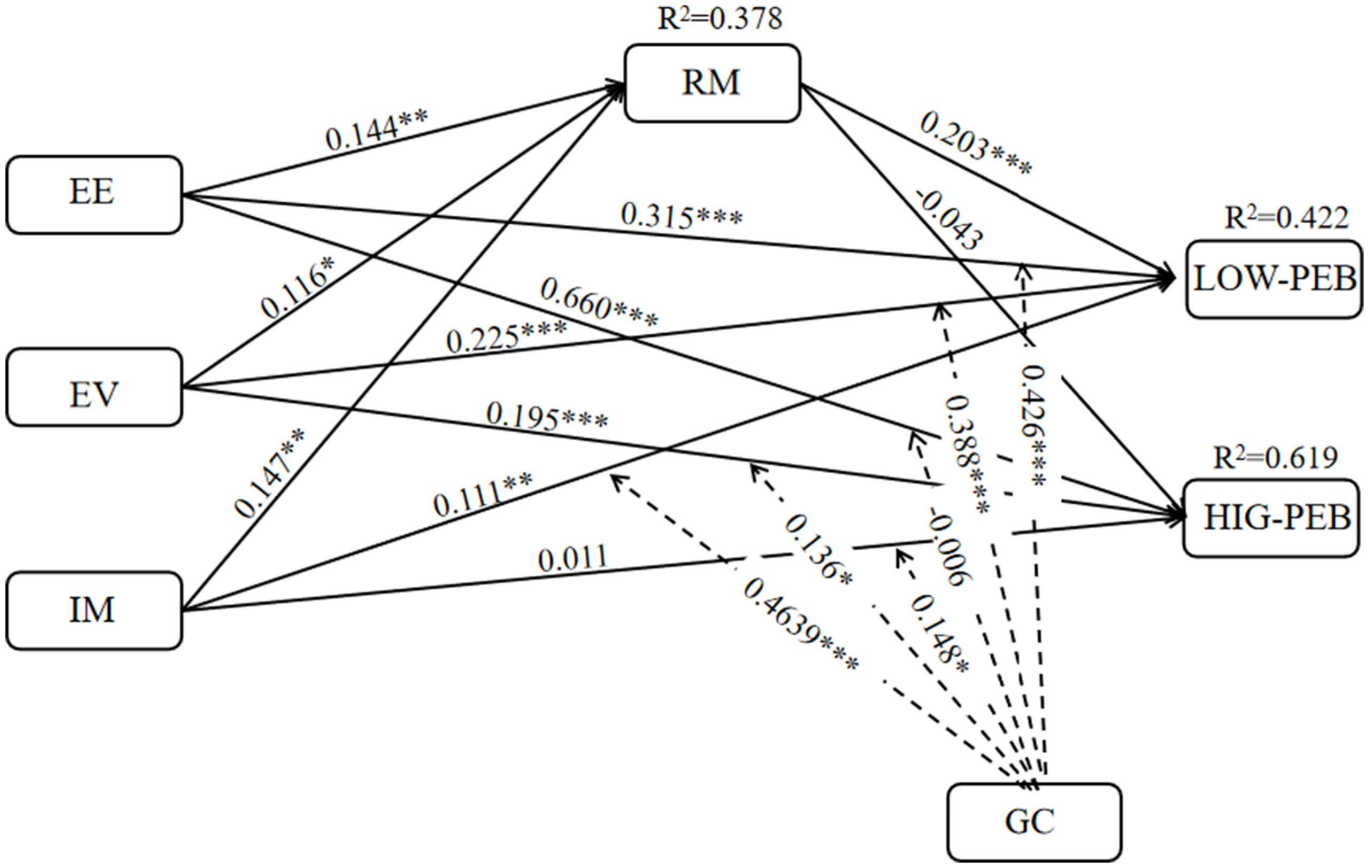
Results of the proposed model.

First, as illustrated in Figure 2 and Table 5, two path coefficients (0.011 and −0.043) were not significant (*p* > 0.05), whereas the remaining path coefficients were significant (*p* < 0.01 or *p* < 0.001). Second, the R^2^ values for the variables are RM = 0.378, LOW-PEB = 0.422, and HIG-PEB = 0.619, indicating that the structural model possesses good explanatory power. Third, we assessed Stone-Geisser’s Q^2^ values, which reflect the predictive relevance of the model. In this study, the Q^2^ values for construct cross-validated redundancy were (Q^2^_LOW-PEB_ = 0.224, Q^2^_HIG-PEB_ = 0.366), both > 0. This indicated that the structural model had sufficient predictive relevance (Urbach and Ahlemann, 2010).

**Table 5:** Structural model fit indices.

	LOW-PEB	IM	EV	EE	RM	HIG-PEB	Model fit indices
*R* ^2^	0.422				0.378	0.619	
*Q* ^2^	0224					0.366	
*f* ^2^							
LOW-PEB							
IM	0.034				0.036	0	
EV	0.045				0.048	0.052	
EE	0.105				0.115	0.699	
RM	0.063					0.004	
HIG-PEB							
SRMR							0.072
d_ULS							0.669
d_G							0.551
NFI							0.926
GOF							0.621

The thresholds for f^2^ values were as follows: 0.02 was considered a small effect, 0.15 a medium effect, and 0.35 a large effect (Cohen, 2013). The values obtained in this study meet these criteria, thus confirming the suitability of the model.

In SmartPLS, the model fit was assessed using three absolute fit indices: SRMR, NFI, and RMStheta. The overall fit metrics were as follows: SRMR = 0.072 (<0.08), d_ULS = 0.669 (<0.95), d_G = 0.551 (<0.95), NFI = 0.926 (>0.9), indicating that the overall model fit was acceptable (Hair et al., 2019). In addition, we calculated the GoF following the guidelines of Tenenhaus et al. (2005) to evaluate the overall quality of the research model. The GoF value was 0.621, which exceeds the threshold of 0.36, as recommended by Wetzels et al. (2009). This finding suggests that the overall quality of the research model was high.

### 4.3 Multicollinearity and common method bias

Harman’s single-factor exploratory factor analysis is commonly used to detect CMB (Fuller et al., 2016). In this study, the percentage of covariance explained by a single factor after unrotated factor analysis was 34.115%, which was below the 50% threshold. This indicates that the influence of CMB was not significant in this study. Multicollinearity is defined as the inverse of tolerance and assessed using the variance inflation factor (VIF) for formative dimensions. Within the context of PLS-SEM, the recommended VIF value (<3.3) was used as the cutoff criterion (Wong, 2013). The VIF values for this study are provided in Table 6, confirming that multicollinearity was within acceptable limits.

**Table 6:** Variance inflation factor.

Path	VIF	Path	VIF	Path	VIF
IM → LOW-PEB	1.544	IM → HIG-PEB	1.544	IM → GC	1.52
EV → LOW-PEB	1.94	EV → HIG-PEB	1.94	EV → GC	1.925
EE → LOW-PEB	1.632	EE → HIG-PEB	1.632	EE → GC	1.609
GC → LOW-PEB	1.129	GC → HIG-PEB	1.129		

### 4.4 Hypothesis testing and validation

We conducted a path analysis of the latent and observed variables within the overall model to verify each research hypothesis (Table 7).

**Table 7:** Analysis of model path coefficients.

Hypotheses	Path	Coefficient	*t*-value	*p*-value	Supported
H1a	EE → RM	0.144	3.068	**	Y
H1b	EE → LOW-PEB	0.315	8.5	***	Y
H1c	EE → HIG-PEB	0.66	18.695	***	Y
H2a	EV → RM	0.116	2.19	*	Y
H2b	EV → LOW-PEB	0.225	5.584	***	Y
H2c	EV → HIG-PEB	0.195	4.932	***	Y
H3a	IM → RM	0.147	3.103	**	Y
H3b	IM → LOW-PEB	0.111	2.789	**	Y
H3c	IM → HIG-PEB	0.011	0.38	0.704	N
H4a	RM → LOW-PEB	0.203	5.231	***	Y
H4b	RM → HIG-PEB	−0.043	1.829	0.067	N

*P < 0.05; **P < 0.01; ***P < 0.001.

As shown in Table 7, hypotheses H4b (*β* = −0.043, *p* > 0.5) and H3c (*β* = 0.011, *p* > 0.5) were not supported. However, hypotheses H1a, H1b, H1c, H4a, H2a, H2b, H2c, H3a, and H3b were validated.

### 4.5 Mediation effect testing

The mediation effects in this study were tested using the bootstrap method, a nonparametric resampling procedure that does not impose distributional requirements on the mediation effects. This method effectively addresses the issue of non-normal distributions. Following the multiple mediation analysis process proposed by Zhang and Kang (2016), we conducted 1,000 resampling iterations with replacement and set a confidence interval of 95%. The results are summarized in Table 8.

**Table 8:** Mediation effect test.

Hypotheses	Path	Coefficient	*P*-value	95% CI	Supported
H5a	IM → RM → HIG-PEB	0.027	**	[0.009, 0.046]	Y
H5b	EV → RM → HIG-PEB	−0.005	0.164	[−0.013, 0.001]	N
H5c	EE → RM → HIG-PEB	−0.006	0.133	[−0.016, 0]	N
H5d	IM → RM → LOW-PEB	0.03	**	[0.011, 0.051]	Y
H5f	EV → RM → LOW-PEB	0.024	*	[0.003, 0.05]	Y
H5e	EE → RM → LOW-PEB	0.029	**	[0.009, 0.052]	Y

*P < 0.05; **P < 0.01; ***P < 0.001.

First, the mediation effect values of RM were 0.027, 0.03, 0.024, and 0.029, with bootstrap confidence intervals of (0.009, 0.046), (0.011, 0.051), (0.003, 0.05), and (0.009, 0.052), respectively. Because the 95% confidence intervals do not include 0, it can be concluded that RM acts as a mediator in the relationships between IM and low-cost PEB; EE and low-cost PEB; EV and low-cost PEB; and IM and high-cost PEB. Therefore, H5a, H5d, H5f, and H5e are supported.

Second, the mediation effect values of RM for the relationships between EE and high-cost PEB and between EV and high-cost PEB were −0.005 and −0.006 with bootstrap confidence intervals of (−0.013, 0.001) and (−0.016, 0), respectively. As these confidence intervals include 0 and p > 0.5, hypotheses H5b and H5c are not supported. This indicates that RM does not mediate the effects of EE on high-cost PEB or EV on high-cost PEB.

### 4.6 Moderation effect test

The moderation effects were analyzed using SPSS PROCESS Model 5 (Table 9), with age and gender as control variables. The results indicate the following: 1. GC moderates the effect of environmental emotion on low-cost PEB (*β* = 0.426, *p* < 0.001), supporting hypothesis H6a. 2. GC moderates the effect of EV on low-cost PEB (*β* = 0.388, *p* < 0.001), supporting hypothesis H6c. 3. GC moderates the effect of EV on high-cost PEB (*β* = 0.136, *p* < 0.05), supporting hypothesis H6d. 4. GC moderates the effect of IM on low-cost PEB (*β* = 0.463, *p* < 0.001), supporting hypothesis H6f. 5. GC moderates the effect of IM on high-cost PEB (*β* = 0.148, *p* < 0.05), supporting hypothesis H6e. 6. However, GC does not moderate the effect of environmental emotion on high-cost PEB (*β* = −0.006, *p* > 0.05), meaning that hypothesis H6b is not supported.

**Table 9:** Results of the moderation analysis.

Hypotheses	Path	Coefficient	*P*-value	95% CI	Supported
H6a	GC × EE → LOW-PEB	0.426	***	[0.248, 0.604]	Y
H6b	GC × EE → HIG-PEB	−0.006	0.901	[−0.105, 0.092]	N
H6c	GC × EV → LOW-PEB	0.388	***	[0.197, 0.579]	Y
H6d	GC × EV → HIG-PEB	0.136	*	[0.0002, 0.272]	Y
H6f	GC × IM → LOW-PEB	0.463	***	[0.271, 0.655]	Y
H6e	GC × IM → HIG-PEB	0.148	*	[0.006, 0.291]	Y

*P < 0.05; **P < 0.01; ***P < 0.001.

## 5 Conclusion and discussion

This study yielded the following conclusions:

1. Significant Positive Effects on Low-Cost PEB: Environmental emotion (H1b), EV (H2b), IM (H3b), and RM (H4a) all have a significant positive impact on low-cost PEB.
2. Positive and Non-Significant Effects on High-Cost PEB: EE (H1c) and EV (H2c) positively influence high-cost PEB. However, IM (H3c) and RM (H4b) have no significant effect on high-cost PEB.
3. Positive Effects on RM: EE (H1a), EV (H2a), and IM (H3a) positively affect RM.
4. Mediating Role of RM: RM mediates the impact of EE (H5f), EV (H5e), and IM (H5d) on low-cost PEB. Additionally, RM mediates the impact of IM (H5a) on high-cost PEB. However, it does not mediate the effects of environmental emotion (H5b) or EV (H5c) on high-cost PEB.
5. Moderation Effects of GC: GC moderates the effects of EE (H6a), EV (H6c), and IM (H6f) on low-cost PEB. It also moderates the effects of EV (H6d) and IM (H6e) on high-cost PEB. However, GC does not moderate the effect of EE (H6b) on high-cost PEB.

Values and personal norms are crucial internal factors that influence PEB (Karp, 1996). In this study, EV significantly affected low-cost (*p* < 0.001) and high-cost (*p* < 0.001) PEBs. One possible explanation is that individuals with high EV are more likely to develop green and low-carbon lifestyles and awareness. Such values and awareness guide their behavior, making them more willing to engage in pro-environmental actions (Chwialkowska et al., 2020). When individual goals are aligned and normative, they can encourage PEB (Steg et al., 2014). However, conflicting goals may decrease willingness to act pro-environmentally. This indicates a significant correlation between goals and values, suggesting that integrating them into a comprehensive framework of PEB can serve as an effective intervention (Uehara and Ynacay-Nye, 2018). This study further confirms that GC moderates the impact of EV on high- and low-cost PEBs. EV influence low-cost PEB through RM but do not affect high-cost behaviors via RM. Related studies suggest that when such behaviors become overly effortful, costly, or unpleasant, the influence of biospheric values and normative factors in predicting them diminishes significantly. For high-cost PEBs (e.g., purchasing eco-friendly products or participating in environmental campaigns), individuals are more likely to rely on rational evaluations, long-term benefits, and social norms, rather than immediate emotional states or RM. For instance, when deciding whether to buy eco-friendly products, consumers may prioritize factors such as price, quality, and sustainability over emotional influences or trust-based relationships (Abrahamse and Steg, 2011; Niu et al., 2025). Another potential explanation is that the mechanism linking RM and EE varies across different types of behaviors. For high-cost PEBs, social identity, group behavior, and cultural or policy-driven guidance may play more significant roles than RM and emotions (Rychlowska et al., 2015). Therefore, further research is required to investigate the driving factors behind different types of PEB and explore the complex interplay between emotions, social relationships, and behavioral costs.

Individuals may engage in pro-environmental actions out of normative considerations to enhance their social status, indicating that PEB can serve instrumental goals. For example, when status motives are activated, individuals are more likely to choose eco-friendly products over more luxurious but non-green options. This tendency becomes particularly pronounced when the price of the eco-friendly option is only slightly higher (rather than significantly lower) than that of the non-green option, and when decisions are made in public rather than private settings (Griskevicius et al., 2010). Venhoeven et al. (2013) suggested that PEBs enhance social status and cause feelings of pleasure. Thus, personal sacrifices made to the environment can sometimes lead to improved psychological well-being rather than deterioration. This demonstrates that IM influences low-cost PEB. Conversely, an individual’s actions play a critical role in the formation of their social capital, as mutual collaboration, participation in activities, and selfless contributions are essential for fostering understanding and trust among individuals (Putnam, 1993). In environments with low social capital in relational dimensions (such as the travel groups in this study) and considering the costs tourists must bear to engage in PEB, individuals may show less willingness to demonstrate selfless IM motives, as these are less likely to yield higher social capital. Conversely, in environments with high relational social capital, individuals are more inclined to exhibit strong IM motives. Consequently, IM motives fail to directly influence individuals’ high-cost PEBs. However, RM mediates the relationship between IM motives and high-cost PEBs. Moreover, according to goal-framing theory (Lindenberg and Steg, 2007), environmental behavior is primarily driven by three different types of goals (or motivations) in specific contexts: hedonic, gain, and normative goals. Hedonic goals prompt individuals to seek ways to improve their immediate feelings, such as avoiding effort, pursuing instant gratification, and seeking excitement. Gain goals make individuals particularly sensitive to changes in personal resources such as money or status enhancement. Normative goals guide people to focus on the appropriateness of their behavior and motivate them to remain highly attentive to fulfilling their perceived responsibilities, such as contributing to environmental protection or demonstrating exemplary behavior. Research indicates that PEBs can satisfy normative, hedonic, and gain goals (Steg et al., 2014). In specific situations, the strongest or most salient goal (the “goal frame”) exerts the most significant influence on cognitive processes and decision-making (Lindenberg, 2012). Therefore, when there is strong GC within a group, it strengthens IM motives, enabling them to influence individuals’ high-cost PEBs.

Emotional variables, as significant factors influencing behavior, have been increasingly emphasized in PEB research (Antonetti and Maklan, 2014). Niu et al. (2025) explored the role of emotions in fostering pro-environmental intentions and actions. Pooley and O’Connor (2000) found that emotions can influence environmental behavior by shaping individuals’ environmental attitudes. However, our study revealed that EE do not influence high-cost PEB through RM. Previous studies concluded that RM can foster positive emotions, thereby establishing trust among groups (Rychlowska et al., 2015). This study found that RM mediates the effect of EE on low-cost PEB; however, this phenomenon does not occur for high-cost PEB. A possible explanation for this is that high-cost PEB often entails significant economic investment, time commitment, or behavioral dedication. Thus, decision-making in such contexts may not be entirely influenced by emotions or interpersonal RM. For high-cost PEBs such as purchasing eco-friendly products or participating in environmental initiatives, individuals are more likely to rely on rational evaluations, long-term benefits, and social norms rather than immediate emotional states or RM. For example, when deciding whether to purchase eco-friendly products, consumers may focus more on price, quality, and sustainability, rather than being directly influenced by emotions or trust-based relationships (Niu et al., 2025). Moreover, high-cost PEBs often require strong social support and collective action, making them more dependent on external social or policy incentives rather than direct emotional impacts (Pooley and O’Connor, 2000). Conversely, EE may play a more significant role in low-cost PEBs such as water conservation or waste sorting, as these behaviors are generally simpler and yield visible results in the short term. In such cases, decisions are likely driven by immediate emotional triggers (Antonetti and Maklan, 2014). Additionally, the relationship between RM and EE may operate through different mechanisms across various types of behaviors. For high-cost PEBs, factors such as social identity, group dynamics, and cultural or policy guidance may be more important than RM and emotions (Rychlowska et al., 2015). Therefore, further research is required to explore the driving factors behind different types of PEBs and examine the complex interplay between emotions, social relationships, and behavioral costs.

RM is considered a key socioecological variable that measures the degree of freedom and opportunities a society provides individuals with to choose and adjust their interpersonal relationships based on their personal preferences (Yuki and Schug, 2020). RM can lead to various psychological and behavioral differences, such as general trust (Thomson et al., 2018), factors determining happiness and mental health (Sato et al., 2014), self-esteem (Falk et al., 2009), and interpersonal relationship quality (Li L. M. W. et al., 2015; Li L. et al., 2015). Previous studies noted that the level of RM significantly influences the process of relationship formation and the structure of social networks (Oishi, 2010). Specifically, compared with low-mobility environments, high-mobility environments tend to foster the formation of broader friendship networks (Lun et al., 2012), reduce individuals’ vigilance toward potential risks in friendships, and lessen unnecessary concerns about the presence of enemies (Li L. M. W. et al., 2015; Li L. et al., 2015). However, this study found that RM does not influence high-cost PEB. A possible explanation is that high-cost PEBs typically involve significant resource investments (e.g., time, money, or long-term commitments), in which the decision-making process is more rational and deliberate, exceeding the socio-ecological effects of RM. Individuals in high-mobility environments may be more inclined to build extensive social networks and exhibit a strong sense of trust (Lun et al., 2012; Thomson et al., 2018). However, this tendency might primarily impact low-cost, easily executable behaviors, and may be insufficient to drive individuals to commit substantial resources to support pro-environmental actions.

High-cost PEBs often require strong intrinsic motivation or a high level of environmental value recognition rather than relying solely on RM in interpersonal interactions. For instance, individuals may weigh the economic returns, time costs, and practical needs of their actions, leading to more in-depth and rational decision-making (Yuki and Schug, 2020). Moreover, although RM can enhance trust among individuals and reduce risk vigilance (Li L. M. W. et al., 2015; Li L. et al., 2015), this trust may not serve as a direct motivator in high-cost decisions as it does in low-cost behaviors. Additionally, high-cost PEBs are more likely to be influenced by long-term factors such as personal values, expectations of future rewards, and social recognition. These factors are typically not easily altered by frequent adjustments to interpersonal relationships (Sato et al., 2014). Thus, although relationships in high-mobility environments may foster social interactions and immediate behavioral responses, individuals are more likely to base their choices on personal beliefs and long-term benefits when it comes to high-cost behaviors rather than depending on the situational support provided by the fluidity of social networks.

## 6 Theoretical contributions

By examining Social Cognitive Theory (SCT) within the distinctive context of temporary tourist groups, this study refines and extends the theory in the following four aspects, thereby advancing its theoretical development in tourism research.

First, it enriches the “environmental” dimension of SCT by incorporating group dynamics. A central contribution of this research lies in its successful operationalization of relational mobility—a construct capturing the dynamic evolution of interpersonal connections within groups—as a crucial “environmental” factor within the SCT framework. This approach transcends the conventional tendency in tourism research to treat environment as a static physical or socio-cultural backdrop. Our empirical evidence confirms that within the transient social formation of temporary tourist groups, the dynamic nature of the interpersonal environment possesses explanatory power comparable to individual cognition in shaping behavior. This expands the scope of SCT’s application in tourism research, enabling it to effectively interpret individual behavioral decisions not only within stable environments but also within dynamic and evolving social micro-environments.

Second, it introduces “behavioral cost” as a critical boundary condition. By empirically demonstrating that distinct mechanisms underlie high-cost versus low-cost pro-environmental behaviors, this study refines SCT’s model of triadic reciprocal determinism. The findings reveal that the interactions among personal, behavioral, and environmental factors described by SCT do not represent a universal principle; rather, their strength and nature are significantly constrained by behavioral cost. This implies that social cognitive mechanisms prominent in low-cost contexts (e.g., impression management) may be superseded by deeper personal value judgments in high-cost situations. Consequently, this research establishes a crucial boundary condition for the SCT model, asserting that future applications of the theory for predicting or interpreting tourist behaviors must incorporate the “behavioral cost” variable, thereby enhancing the theory’s predictive precision.

Third, it clarifies the operational pathways and scope of socio-cognitive mechanisms. This research has not only verified the mediating role of relational mobility within the SCT framework but, more significantly, has deepened our understanding of SCT’s operational mechanisms by revealing a “mechanism dissociation” in this mediating pathway across high- and low-cost behaviors. It clearly delineates the respective explanatory domains of social dynamic mechanisms (relational mobility) and intrinsic personal mechanisms (values): the former primarily governs low-cost, highly socially visible behaviors, while the latter dominates high-cost, low social visibility behaviors. This finding advances SCT from a relatively generalized framework toward a more refined theoretical model capable of explaining multiple, parallel behavioral pathways.

Finally, it delineates the applicability boundaries of impression management motivation. This study provides a crucial theoretical qualification to impression management—a key motivational factor within SCT. The findings indicate that the driving efficacy of impression management as a socio-cognitive motivation is strictly confined to the realm of low-cost behaviors. This directly challenges any implicit assumption within tourism research that social image serves as a universal driver. It clearly argues that for high-cost environmental actions, the theoretical focus must shift from socio-cognitive motivations to moral and value-based motivations. This insight will assist future research in applying SCT by enabling a more precise selection of explanatory variables tailored to different behavioral types.

## 7 Practical implications

Based on the specific findings derived from the context of natural reserve settings, this study offers the following targeted practical implications for managers of similar tourism destinations. These recommendations are designed to leverage group dynamics to more effectively promote tourists’ pro-environmental behaviors (PEB).

First, the results clearly demonstrate that low-cost and high-cost pro-environmental behaviors are driven by distinct factors. Hence, management interventions must be highly targeted. Specifically, leverage social motivations and situational convenience to promote low-cost PEB. Given the significant influence of relational mobility and impression management on low-cost behaviors, management strategies should focus on making environmentally friendly actions “socially desirable” and easy to perform. For instance, scenic areas can establish interactive and engaging check-in points for environmental protection (e.g., uniquely designed sorting bins) to encourage tourists to share their eco-friendly moments on social media, thereby satisfying their impression management needs. Simultaneously, tour guides should proactively foster a consensus on environmental protection within the group, framing the practice of low-cost PEBs (such as carrying out one’s trash) as a “new trend” that gains group recognition.

Second, this study reveals that in high-cost behavioral contexts, intrinsic environmental values and group-level goal congruence become paramount. Therefore, for behaviors requiring significant tourist investments of time, money, or convenience (e.g., participating in additional ecological restoration activities, opting for higher-priced eco-friendly products), promotional and interpretive efforts should prioritize profound value-based communication, clearly articulating their long-term environmental benefits. Moreover, tourism managers (such as tour guides or group leaders) should strategically cultivate and reinforce collective environmental goals during the initial formation of the group. Leveraging this goal congruence can effectively foster members’ commitment to high-cost environmental actions.

Third, this study reveals that the internal social dynamics of temporary tourist groups represent an actionable lever for interventions. Reposition tour guides as “facilitators of a pro-environmental atmosphere.” Training programs for guides should be enhanced, enabling them to transcend their traditional role as narrators and become active shapers of the group’s environmental culture. This training should equip them with skills to rapidly foster connections among group members (thereby enhancing relational mobility) and to adeptly integrate environmental goals into the group’s agenda, consistently reinforcing them to leverage goal congruence for promoting pro-environmental behaviors.

Finally, it is crucial to prevent pro-environmental actions from becoming isolated individual endeavors. Managers can design environmental activities that require small-group collaboration—such as team-based competitions for nature observation documentation or area clean-up challenges—thereby seamlessly embedding pro-environmental behaviors within social interactions and effectively harnessing group dynamics.

## 8 Research limitations and directions for future study

This study contributes to the understanding of the factors influencing PEB, but also has certain limitations that provide opportunities for further exploration. The following outlines the limitations and directions for future research. First, the sample of this study was primarily drawn from Jiuzhaigou Valley National Nature Reserve, which may limit the generalizability of the findings. Typically, tourists visiting nature reserves may possess a higher baseline level of environmental values, and those with higher education levels often demonstrate more positive environmental awareness and attitudes. Compared to visitors to urban or cultural heritage sites, this segment may exhibit greater inherent concern for the environment. Consequently, the generalizability of our results across diverse cultural contexts and tourism settings requires further verification. It is therefore recommended that future research collect data from various tourism contexts (e.g., urban leisure, beach resorts) to explore how environmental emotions, environmental values, relational mobility, and impression management influence pro-environmental behaviors across different settings, thereby enhancing the external validity of the findings.

Second, insufficient differentiation between high-cost and low-cost behaviors. This study differentiates between high- and low-cost PEBs; however, the boundary between the two may be somewhat ambiguous, and the definition of behavioral cost is subjective. For instance, individuals may perceive “high cost” or “low cost” differently based on their economic status, time constraints, and social standing. Future research could refine the classification of high- and low-cost behaviors and explore how different types of behaviors are influenced by factors such as emotions, relationship fluidity, and goal consistency. Additionally, incorporating objective measures of behavioral costs, such as financial expenses and time consumption, could enhance the precision of the study.

Third, limitations regarding causal inferences inherent to cross-sectional research. The present study utilized cross-sectional data to examine the effects of environmental emotion, relational mobility, and impression management on pro-environmental behaviors. However, this design precludes definitive causal inferences. Furthermore, the use of self-reported measures may introduce social desirability bias. Therefore, it is recommended that future research employ longitudinal designs and incorporate diverse methodological approaches—such as observational data and objective behavioral indicators—to enhance methodological rigor and better capture the dynamic relationships and causal pathways among variables. For instance, subsequent studies could track individuals over time to examine how fluctuations in emotions, adjustments in relational mobility, and the evolution of values influence the long-term development of pro-environmental behaviors.

Fourth, limited exploration of sociocultural factors. This study primarily focuses on individual-level factors, whereas the role of sociocultural factors, such as cultural background, social norms, and policy environments, has not been fully examined. Determinants of PEB may vary across cultures. For instance, IM may play a more prominent role in cultures with high relational fluidity or emphasis on individualism. Future research could analyze how sociocultural factors moderate PEBs in cross-cultural contexts and investigate how these cultural elements influence intentions and actual behaviors. These findings can inform environmental policies and promotional strategies tailored to diverse cultural settings.

Fifth, further exploration of the relationship between emotions and behavioral costs. This study found that emotions play a more significant role in low-cost PEBs and have a weaker influence on high-cost behaviors. Future research could delve deeper into the mechanisms by which different types of emotions (e.g., positive versus negative emotions) impact high- and low-cost behaviors. Additionally, studies could explore how emotions interact with other factors such as personal beliefs and external incentives in more complex scenarios. For instance, negative emotions (e.g., anger over environmental degradation) may strongly drive high-cost PEBs in specific contexts. Various experimental designs can be used to investigate these mechanisms in detail.

## Data Availability

The data analyzed in this study is subject to the following licenses/restrictions: The datasets generated and/or analyzed during the current study are not publicly available due to participant privacy and ethical restrictions but are available from the corresponding author on reasonable request. Requests to access these datasets should be directed to 181457527@qq.com.
